# Acceptability and safety of a probiotic beverage supplementation (Bio-K +) and feasibility of the proposed protocol in children with a diagnosis of autism spectrum disorder

**DOI:** 10.1186/s11689-025-09617-5

**Published:** 2025-05-24

**Authors:** Ghizlane Gaougaou, Riham Zahra, Sophia Morel, Véronique Bélanger, Inga Sophia Knoth, Dominique Cousineau, Baudouin Forgeot D’Arc, Kelly Grzywacz, Guy Rousseau, Eric Déziel, Roger Godbout, Sarah Lippé, Mathieu Millette, Valérie Marcil

**Affiliations:** 1 Department of Nutrition, Université de Montréal, Montreal, QC H3 T 1 A8 Canada; 2 Bio-K Plus International Inc., Kerry (Canada) Inc., 495 Armand-Frappier Boulevard, Laval, QC H7 V 4B3 Canada; 3 Department of Psychology, Université de Montréal, Montreal, QC H3 T 1 A8 Canada; 4 Department of Pediatrics, Université de Montréal, Montreal, QC H3 T 1 C5 Canada; 5 Department of Psychiatry, Université de Montréal, Montréal, QC H3 C 3 J7 Canada; 6 Division of Gastroenterology, Hepatology and Nutrition, Department of Pediatrics, CHU Sainte-Justine, Montreal, QC H3 T 1 C5 Canada; 7 Research Center, Centre Intégré Universitaire du Nord-de-L’Île-de-Montréal, Montreal, QC H1E 1 A4 Canada; 8 Department of Pharmacology and Physiology, Université de Montréal, Montreal, QC H3 C 3 J7 Canada; 9 Centre Armand-Frappier Santé Biotechnologie, Institut National de La Recherche Scientifique (INRS), Laval, QC H7 V 1B7 Canada; 10 Centre de recherche Azrieli du CHU Sainte-Justine, 3175 Côte Sainte-Catherine, Montreal, QC H3 T 1 C5 Canada

**Keywords:** Autism spectrum disorders, Probiotics, Acceptability, Safety, Feasibility, Children, Autistic symptoms, Gastrointestinal symptoms, Sleep disorder

## Abstract

**Background:**

Autism spectrum disorder (ASD) is a group of neurodevelopmental disorders defined by stereotyped behavior and challenges in social communication and social interaction. ASD is associated with various comorbidities, including anxiety, gastrointestinal (GI) symptoms and sleep disorders. Evidence supports an association between intestinal dysbiosis and the severity of ASD-related symptoms. Probiotic intake was suggested to restore microbial homeostasis and decrease neurobehavioral, GI and sleep symptoms in individuals diagnosed with autism.

**Methods:**

This study aims to evaluate the acceptability and safety of a Bio-K + probiotics beverage in autistic children aged 4 to 11 years and the feasibility of the proposed research protocol to measure its impact on behaviors and comorbidities. The 30-week study consisted of daily supplementation with Bio-K + probiotics for 14 weeks. Acceptability and safety were monitored throughout the study. Feasibility was assessed by comparing recruitment and completion rates to pre-established thresholds. Preliminary impact of supplementation on behaviors (Autism Treatment Evaluation Checklist (ATEC) score), GI symptoms and sleep disorders was evaluated.

**Results:**

Of the 23 children recruited (mean age 6.7 ± 2.2 years, 70% males), 65% had GI problems and 91% had sleep disorders. Probiotic supplementation was accepted by all participants and no product-related adverse event was reported. Feasibility rates exceeded pre-established thresholds for almost all study outcomes including recruitment rate, compliance, electroencephalography, actigraphy and completion of questionnaires. Preliminary data suggest an improvement in behaviors associated with autism assessed with the total ATEC score, and in GI symptoms and sleep disorders.

**Conclusion:**

This study demonstrates probiotic beverage acceptability and safety and protocol feasibility in autistic children. To further support our data, a double-blinded placebo-controlled study is needed to determine its efficacy.

**Supplementary Information:**

The online version contains supplementary material available at 10.1186/s11689-025-09617-5.

## Background

Autism spectrum disorder (ASD) is a group of neurodevelopmental disorders characterized by stereotyped behavior and deficits in communication and social interaction [1]. Worldwide, the prevalence of ASD is estimated to be between 1 and 2% [2]. In Canada, 1 in 50 (2%) children and youth aged 1 to 17 years are diagnosed with ASD [3]. While the exact etiology of ASD remains unknown, several studies have suggested a complex interaction between genetic, epigenetic, and environmental factors including a possible role for the gut microbiota [4–8]. Clinical features of ASD include the impairment of communication abilities and social development, the presence of repetitive/restrictive behaviors [9, 10], language delay, learning disabilities and challenges with social interactions (reviewed in [11–13]). ASD can be associated with a large variety of developmental and mental condition, including intellectual impairment, attention-deficit hyperactivity disorder (ADHD), obsessive–compulsive disorder and anxiety [14], epilepsy [15] and intellectual disability [16, 17].

Current management of ASD includes behavioral interventions such as applied behavior analysis (ABA) [18], speech therapy (reviewed in [19]), occupational therapy [20], and pharmacotherapy for associated symptoms. Comprehensive interventions with active caregiver involvement such as ABA can help to enhance effective communication, social interaction, behavior, and independence [21]. However, they often rely heavily on the services of professionals who can be difficult to access. Also, these interventions add to the already heavy burden on families [22–25] and the conclusions about their effectiveness may be limited by their experimental design as many studies regarding ABA interventions are not randomized controlled trial (RCT) (reviewed in [26]).

Other conditions are commonly associated with ASD which can affect quality of life of children and caregivers, but they receive limited recognition and care [22–25, 27]. A high prevalence of sleep problems has been reported in ASD [28, 29], with children experiencing sleep disturbances and circadian sleep alterations including sleep resistance, prolonged sleep onset, long or frequent nocturnal awakenings and early morning awakenings [30–32]. In addition to impairing quality of life [33, 34], these problems could alter brain development and function [28, 35].

Moreover, gastrointestinal (GI) disorders such as abdominal pain, diarrhea, constipation, and bloating have been reported in proportions as high as 91% in ASD [36–41]. In some children with ASD, abdominal pain may be expressed through aggression or self-mutilation given their incapacity to communicate their pain or frustration effectively ([42], reviewed in [43]). Other children may become more restless or irritable, withdraw or isolate themselves [44–46]. GI disorders can also increase anxiety and emotional dysregulation, which can lead to tantrums or high anxiety levels [46, 47]. These GI disorders can cause sleep disturbance, which can exacerbate behavioral problems and negatively impact the quality of life [46, 47]. Emerging data suggest that gut dysbiosis may be associated to ASD and its symptoms ([48], reviewed in [49]), suggesting a bidirectional communication through the gut-brain axis. While an association between an altered gut microbiota and autistic behaviors has been described ([50–59], reviewed in [60]), it remains controversial and without proof of cause to effect relationship [61, 62].

Acknowledging a possible role for the gut microbiota in ASD symptomology, interventions acting on its modulation have been proposed, such as prebiotics, probiotics and microbial transfer therapy (MTT) [63], reviewed in [64, 65]). Probiotic intake was suggested to restore microbial homeostasis and decrease neurobehavioral, GI and sleep symptoms in individuals diagnosed with autism (reviewed in [64]). In contrast, other studies failed at demonstrating an impact of probiotics on autistic behaviors [66, 67]. These conflictual results suggest that the possible efficacy of probiotic supplementation could be related to the bacterial strains used and other factors influencing outcomes, including the study design, population, intervention period, and placebo effect. Consequently, there is a need for well-designed clinical studies to establish a solid ground for the use of probiotics within ASD populations.

Bio-K + is a commercially available and well-defined specific probiotic with a patented formulation containing a combination of 3 bacterial stains, *Lactobacillus acidophilus* CL1285, *Lactobacillus casei* LBC80R and *Lactobacillus rhamnosus* CLR2. These specific strains prevent and reduce the severity of antibiotic-associated diarrhea [68] and particularly reduce *Clostridioides difficile*-associated diarrhea in patients receiving antibiotic therapy in hospitals [69–71]. A study highlighted that this specific probiotic combination improves the symptoms and quality of life of individuals affected by the irritable bowel syndrome [72].

The aims of this study were to test the acceptability and the safety of the probiotic beverage supplement (Bio-K +) and the feasibility of the proposed protocol in a pediatric population with a diagnosis of ASD. As a complementary objective, preliminary data were collected to assess the impact of the probiotic beverage supplementation on behaviors, as well as sleep and GI symptoms.

## Methods

### Participants and eligibility criteria

Participants were recruited from September 2021 to December 2021 by response to an advertisement posted on the CHU Sainte-Justine website, Facebook, page, and Twitter (now X) account, and from April 2022 to June 2022 through consultation of medical charts of children diagnosed with ASD followed at the Integrated Center for Child Neurodevelopment Network (CIRENE) of the CHU Sainte-Justine. To be eligible, the identified children had to meet the following inclusion criteria: (1) medical diagnosis of ASD; (2) age between 4–11 years old; (3) acceptance and ability to consume the probiotic beverage for the duration of the study. Exclusion criteria were: (1) autism in the context of a genetic syndrome such as fragile X or tuberous sclerosis complex; (2) cancer, diabetes or genetic disorder such as Down syndrome 21 or 14; (3) immune system disorder; (4) intolerance or allergy to the probiotic beverage; (5) having taken probiotics during the previous 3 months and; (6) having taken antibiotics in the previous month. All parents provided informed consent.

### Study design

**Fig. 1:**
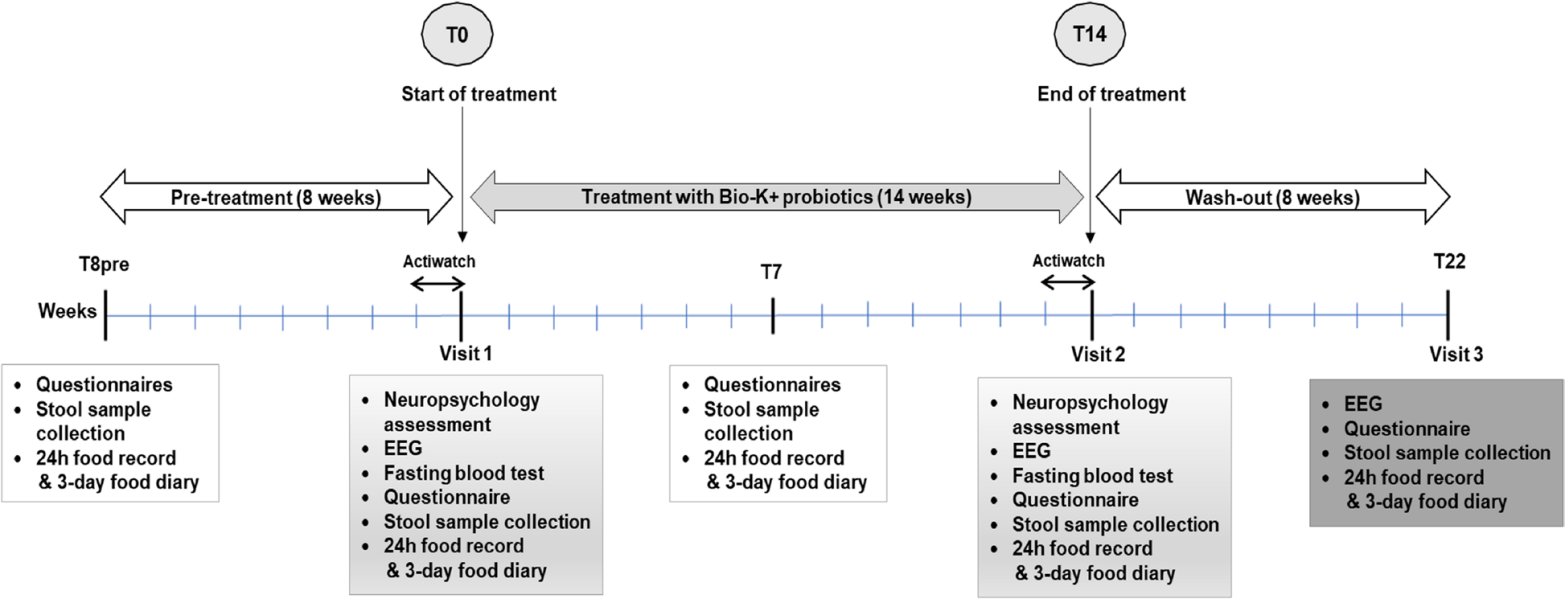
Study design. The participation period comprised 3 phases: (1) pre-treatment phase (8 weeks), (2) treatment phase during which the participant receives pea-based, raspberry-flavor probiotics drink (Bio-K +) daily for 14 weeks, (3) wash-out phase (8 weeks). Questionnaires were completed at 5 time-points (T-8pre, T0, T7, T14 and T22); Neuropsychology assessment and blood tests were performed at T0 and T14, EEG was performed at T0, T14 and T22. During the week preceding the T0 and T14 visits, the participant wore an actigraph on the wrist 24 h a day

The study was a 30-week, open label, non-randomized, safety and feasibility trial. The sample size for statistical threshold was not calculated, and withdrawn participants and dropouts were not replaced. The recruitment of 30 participants was initially planned. The study comprised 3 phases, and data were collected at 5 time-points (Fig. 1).

In the pre-treatment phase, data were collected 8 weeks before initiating the probiotic supplementation (T-8pre) via meetings with parents (virtual or in-person). The aim was to validate symptoms stability before initiating treatment. During the treatment phase, children were given the probiotic beverage for daily consumption for 14 weeks. Data were collected at 3 time-points: at baseline (T0) and after 14 weeks (T14), assessments included the completion of questionnaires and a visit to the CHU Sainte-Justine to complete testing; and at mid-treatment (7 weeks, T7), data were collected with questionnaires in a virtual meeting. The third phase consisted of an 8-week wash-out period (T22) to evaluate the persistence of symptoms after probiotic cessation in an assessment that required a visit to the CHU Sainte-Justine.

### Bio-K + Probiotic acceptability and safety

The Bio-K + probiotic supplement consisted of a vegan pea-based, raspberry-flavored fermented drinkable product containing a minimum of 50 × 10^9^ colony forming units (CFU) of three strains: *L. acidophilus* CL1285, *L*. *casei* LBC80R and *L*. *rhamnosus* CLR2. The product was manufactured and graciously supplied by Kerry Canada inc. (Laval, Quebec, Canada). For the duration of the supplementation period, children had to consume 1 bottle per day (98 g or 98 mL) all at once or over the course of the day. Children were allowed to mix the content of the probiotic beverage bottle with any cold beverage.

To assess acceptability and safety, parents were contacted by phone on 6 occasions during the supplementation period (after 1 and 3 days and after 4, 7, 11 and 14 weeks of supplementation) to report on their child acceptance of the product taste (yes/no) and on potential adverse events related to probiotics consumption namely bloating/gas, diarrhea, constipation, abdominal pain, urticaria and vomiting [73, 74]. Parents were instructed to immediately stop probiotic consumption and notify the research team if an adverse event such as diarrhea, vomiting or urticaria or if bloating/gas, constipation, and stomachache persisted for more than 3 days. Product taste was considered acceptable if more than 50% of parents answer"yes"at each phone follow-up. The probiotic beverage was considered not safe if more than 40% of participants reported an adverse effect requiring discontinuation of product during the 14 weeks of supplementation.

### Testing and data collection

#### Assessment of autistic and gastrointestinal symptoms and of sleep

At the 5 time-points, 6 standardized questionnaires were used to assess autistic symptoms, social communication, GI symptoms and sleep. The autism treatment evaluation checklist (ATEC) is a questionnaire designed to assess changes in ASD-related behaviors in individuals under the age of 18-year-old diagnosed with ASD in response to a treatment [75–77]. It includes 77 items summed as a total score (0–179) that are divided in 4 subsections: (1) speech/language/communication (14 items; score range from 0 to 28); (2) sociability (20 items; score range from 0 to 40); (3) sensory/cognitive awareness (18 items; score range from 0 to 36); and (4) health/physical/behavior (25 items; score range from 0 to 75) [77]. A higher total ATEC score indicates greater difficulties or severity thus a reduction in the ATEC score represents an improvement. Participants were classified with mild (score 20–49), moderate (score 50–79) and severe (score > 80) ASD as proposed by Mahapatra et al*.* [78]. The behavior rating inventory of executive function (BRIEF) questionnaire is designed to assess executive functions in home and school environments of children with learning and attention disorders, developmental disabilities, depression and other developmental and neurological disorders (Score: 0–100) [79]. The social communication questionnaire (SCQ) is used to measure social communication and existing symptoms of ASD (score: 0–19) [80].

The gastrointestinal severity index (GSI) quantifies the severity of GI symptoms by attributing a score (0–2) related to 9 components (constipation, diarrhea, stool consistency, stool smell, flatulence, abdominal pain, unexplained daytime irritability, nighttime awakening, abdominal tenderness). The total score ranges between 0–18, with higher values corresponding to greater severity and a score ≥ 4 indicating severe GI disorders [81]. Constipation was identified in participants having 3–4 stools/week (mild/moderate constipation) and 0–2 stools/week (severe constipation) based on the first question of the GSI questionnaire. Participants reporting ^3^5 stools per week were considered not constipated [81].

For sleep evaluation, 2 questionnaires were used: the children’s sleep habit questionnaire (CSHQ) and usual sleep schedule. The CSHQ (total score: 33–99) assesses sleep complaints using eight subscales: (1) bedtime resistance, (2) time to sleep, (3) sleep duration, (4) anxiety at sleep onset, (5) nocturnal awakenings, (6) sleep behaviors, (7) breathing and (8) sleepiness. A CSHQ total score of 41 or more indicates that symptoms are clinically significant and may reflect a sleep disorder [82]. The usual sleep schedule (total score: 0–30) quantifies the number of hours of sleep [30].

#### Neuropsychological evaluation

The neuropsychological evaluation was conducted by a trained neuropsychologist at T0 and T14 using the WISC-V (fifth edition) for children aged 6 years to 11 years and 11 months or the WISC-IV (fourth edition) for children under 6 years old [83–85]. The neuropsychological evaluation was offered in 3 languages according to the preferences of the participants and/or their parents: French, English, or Spanish. Following the completion of these test batteries, a score was attributed to participants and intelligence was classified as extremely high (score ≥ 130); very high (score 120–129); high average (score 110–119); average (score 90–109); low average (score 80–89); very low (score 70–79); extremely low (score 50–69) [83–85].

#### Dietary intake

Participants’ dietary intake was documented prior to each stool sample collection (5 times during the study) to determine if there were significant changes in diet over the course of the trial using 3-day food records and 24-h recalls (24-HR). Both tools were used to calculate energy and nutrient intake by using the web application Nutrific® designed by the Department of food science and nutrition, Université Laval (https://nutrific.fsaa.ulaval.ca, last accessed on April 8 2023) based on the 2015 Canadian nutrient file.

#### Blood tests

Blood samples were collected after a 3 to 4 h fast (9 mL total) at visits T0 and T14. Prior and with the consent of the parent, an anesthetic cream (Maxilene 4®, RGR Pharma LTD, Ontario, Canada) was applied topically to the child’s arm.

#### Stool sample collection

With a view to studying the impact of probiotics on gut microbiota and on its metabolites, stool samples were collected by the parent at home, 1 to 3 days before each time-point (T8-pre, T0, T7, T14 and T22). Parents were instructed to freeze the stool sample (−20 °C) as soon as it was collected and until the delivery at the research laboratory. Detailed instructions were provided on self-collection, packaging and handling of samples and delivery. Upon receipt, samples were stored at −80 °C. At the time of collection, parents were asked to complete the Bristol scale to classify stool type and shape. 

#### Electroencephalography

Electroencephalography (EEG) tests were performed at T0, T14 and T22 to measure to extract signal features relevant to the four tasks. These include event-related components, spectral density, and non-linear features at every electrode site. During the EEG procedure, the child had to wear a 128-channel electrical geodesics incorporated system (Magstim, Eugene, OR, USA). The duration of each EEG test was 45 min including net installation. The EEG protocol included four tasks: resting state, face processing using event-related potentials (ERP), visual steady-state and auditory steady-state. Continuous EEG resting state was recorded until a total of three minutes of clean signal was obtained (i.e. without movement, muscular or other artefacts). Participants watched a video of an abstract moving shape (rather than a fixation cross) to increase compliance and reduce movement during resting state recording. The face ERP task lasted six minutes during which upright and inverted faces and houses were presented. The visual steady-state task lasted five minutes and consisted of 18 colored icons that appeared/disappeared at the center of the screen at frequencies of 6 Hz, 10 Hz, or 15 Hz. Each bloc presented six trials which contained every frequency twice and a total of nine blocs was presented. One hundred auditory steady-state task was performed during the viewing of a muted movie without the soundtrack. Auditory stimulation blocks of 6 Hz or 40 Hz were presented in random order for six minutes [86].

#### Actigraphy

To monitor sleep–wake cycles and possible changes in sleep patterns, children were asked to wear an actigraph 24 h per day for 7 days, before the T0 and T14 visits. This wearable device actiwatch (Actiwatch 64, Mini Mitter Co, Philips Respironics) tracks movements during sleep and awake periods and collects data over an extended time period, providing an objective measurement for the evaluation of sleep disorders [10].

#### Study protocol feasibility

All feasibility outcomes were calculated at the end of the study and include: recruitment rate (% patients approached/participants recruited), retention rate (% participants retained/participants recruited), compliance rate (% patients who consumed the probiotic as expected/participants exposed to supplementation), study data completion rate [sample collection (blood and stool), EEG test, questionnaires, and neuropsychological evaluation] and completion rate of actigraphy measurement. The compliance rate was expressed as percentage of patients compliant to probiotic beverage supplementation according to total participants having started the supplementation phase. To be considered compliant, participants had to consume the probiotic product at least 6 out of 7 days per week for the entire supplementation period (14 weeks). The consumption rate for each participant was also calculated, corresponding to the number of bottles consumed divided by the expected number of bottles (i.e. planned) and is presented as group average. The completion rate of study data represents the sum of each completed study data for all participants over the course of the study (i.e. completed), whereas expected data are based on the total number of measures planned during the study according to available participants (i.e. those still included in the study at the time of data collection). Actigraphy test was considered completed if the participant had worn the actigraph for 24 h for 7 consecutive days. The actigraphy completion rate was calculated as the total number of completed measurements divided by the total number of planned measurements. Therefore, if the participant did not comply with wearing the actigraph for the entire 7-day period the test was considered not completed.

Pre-defined feasibility criteria were adapted from *Mengoni *et al*.* [58] and were: (1) recruitment rate > 40%; (2) retention rate > 35%; (3) compliance rate > 50%; (4) completion rate > 50% for blood sample collection, stool sample collection, EEG test and actigraphy assessment; (5) mean questionnaires completion rate > 80% and; (6) completion rate of neuropsychological evaluation > 80%.

### Data analysis

Description of participants’ characteristics at recruitment is computed as mean ± standard deviation (SD) for continuous variables (age at recruitment) and as percentage (%) of total participants for categorial variables (sex, sibling, non-verbal, GI problems, sleep problems, ADHD, epilepsy, and brain abnormality). For acceptability, safety and feasibility data, the descriptive statistics of variables are presented as percentage (%). The mean questionnaire completion rate is based on the average completion rate of the 8 questionnaires (ATEC, SCQ, BRIEF, GI, CSHQ, usual sleep schedules, 3-day food records and 24-HR). Preliminary assessment of supplementation effect was conducted using scores derived from the ATEC (total and each of the four subscales), the GSI and the CSHQ questionnaires at each time-point which are presented as mean ± standard error (SE). The change in total and each subscale score for ATEC and CSHQ questionnaires during the study were assessed using repeated measures analysis of variance (ANOVA) and Bonferroni correction was applied for multiple testing in *post-hoc* pairwise comparisons. Changes in GSI score overtime were assessed using Friedman test followed by Wilcoxon test as *post-hoc* analysis with Bonferroni correction for multiple comparisons. To further describe the variation of ATEC and GSI scores during the study, the percentage of change in both scores from T0 was calculated for T7, T14 and T22 using T0 scores (i.e. at baseline/before treatment) as 100% for each participant. The proportion of participants according to ASD-related behavior severity (mild/moderate/severe) and severe GI problems (yes/no) is presented as a percentage (%) at each time-points. The proportion of participants with severe autistic behaviors (“severe” vs. “mild and moderate”) and with severe GI problems (yes vs. no) was compared across all time-points using the Cochran Q test, and *post-hoc* analysis was conducted for pairwise comparison between time points using McNemar test with false discovery rate correction for multiple testing.

## Results

### Recruitment of participants and cohort description

**Fig. 2:**
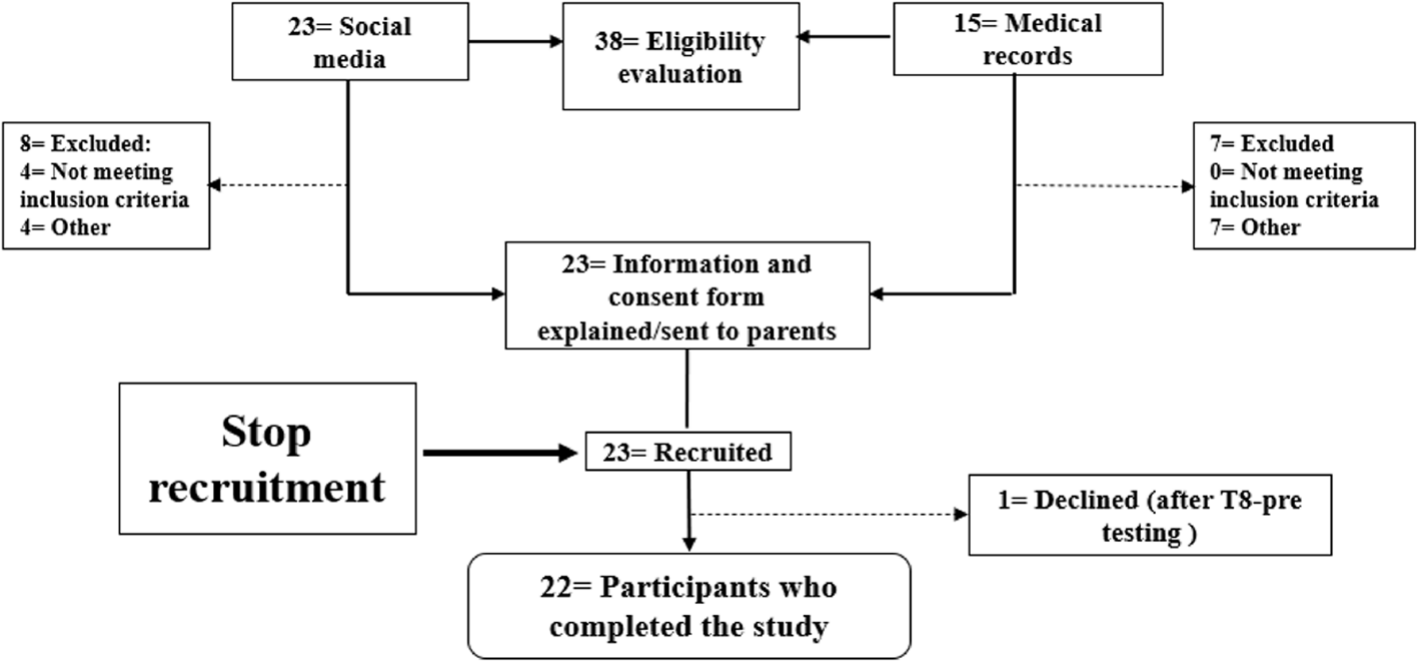
Recruitment of participants in the PRIOBI-O-TISM Study

Thirty-eight participants were evaluated for eligibility according to the inclusion and exclusion criteria (Fig. 2).

A total of 23 parents expressed interest in enrolling their children in the study in response to website and social media postings. Of these, 15 participants were retained, including 14 males and 1 female. The other 7 children were not enrolled for not meeting the inclusion criteria for age (*N* = 4: older than 12 years), and difficulty to commute to the CHU Sainte-Justine (*N* = 4). To increase the number of females in the cohort, recruitment via medical charts was performed. Out of 15 identified and contacted participants, 8 were recruited including 6 females and 2 males. The reasons for the refusal of 7 parents to include their child in the study were: lack of financial compensation (*N* = 2), weekday testing/lack of time (*N* = 4) and difficulty to commute (*N* = 1). The recruitment rate through both methods was 60.5%. Thus, of the 23 children enrolled, 65.2% (*N* = 15) were recruited via social networks and 34.8% via medical charts. While the recruitment of 30 participants was initially planned, only 23 participants were recruited (with the approval of the Data and Safety Monitoring Board (DSMB)) given that study objectives were achieved at that point and that pursuing recruitment was deemed futile.

Demographic characteristics of the recruited participants are described in Table 1.

**Table 1:** Participants’ characteristics and demographic data at recruitment

Characteristics (*N* = 23)	N (%)
Sex	
Male	16 (69.6)
Female	7 (30.4)
Age at recruitment (years), mean ± SD	6.7 ± 2.2
Autistic siblings	4 (17.4)
Non-verbal	7 (30.4)
GI problems^a^	15 (65.2)
Diarrhea	5 (21.7)
Constipation	15 (65.2)
Sleep problems^b^	21 (91.3)
ADHD	4 (17.4)
Epilepsy	1 (4.5)
Brain abnormality	3 (13.6)
Celiac disease	1 (4.5)
Under gluten and casein free diet	3 (13.6)

^a^Participant has at least 1 gastrointestinal problem according to GSI questionnaire

^b^Participants are considered to have sleep problems at a CSHQ score ≥ 41

*SD* standard deviation, *ATEC* Autism Treatment Evaluation Checklist, *ADHD* attention-deficit hyperactivity disorder, *GI* gastrointestinal

The mean age at recruitment was 6.7 years (range of 4.0 to 11.1 years) with 69.6% males (*N* = 16). A total of 5 siblings (21.7%) were included in the study. Of the 23 ASD participants, 7 were non-verbal (30.4%), 1 had epilepsy (4.5%), 4 had ADHD (17.4%), 1 had celiac disease (4.5%) and 2 followed a gluten and casein free diet (8.7%). At the first assessment (T8-pre), severe GI disorders were found in 65.2% of participants (*N* = 15) and severe sleep disturbances in 91.3% (*N* = 21). According to the neuropsychological assessment, 66.7% of participants (*N* = 10) had extremely low intelligence, 26.7% (*N* = 4) average and 6.6% (*N* = 1) low intelligence (Table 2).

**Table 2:** Autistic and gastrointestinal symptoms at study baseline and evaluation of sex differences

Symptoms		Sex		
	All	Females	Males	*P*-value^a^
*Autistic*	*N* = 23	*N* = 7	*N* = 16	
ATEC score, mean ± SD				
Total	59.3 ± 29.6	61.0 ± 41.2	58.5 ± 24.6	0.83
i. Speech/language communication	10.5 ± 9.5	13.9 ± 10.3	9.0 ± 9.0	0.28
ii. Sociability	12.7 ± 8.0	12.6 ± 10.3	12.8 ± 6.7	0.57
iii. Sensory/cognitive awareness	10.4 ± 7.1	11.1 ± 9.6	10.1 ± 6.1	0.91
iv. Health/physical/behavior	25.6 ± 11.3	23.4 ± 13.2	26.6 ± 10.7	0.55
Autistic behaviors severity^b^, N (%)				1.00
Mild	10 (43.5)	3 (42.9)	7 (43.8)	
Moderate	8 (34.8)	2 (28.6)	6 (37.5)	
Severe	5 (21.7)	2 (28.6)	3 (18.8)	
*Gastrointestinal*	*N* = 23	*N* = 7	*N* = 16	
GSI Score, mean ± SD	4.0 ± 2.7	2.6 ± 2.1	4.6 ± 2.8	0.10
Severe GI problems^c^, N (%)				0.19
Yes	12 (52.2)	2 (28.6)	10 (62.5)	
*Neuropsychological*	*N* = 15	*N* = 2	*N* = 13	
Intelligence scale^d^, N (%)				0.50
Extremely high	0	0	0	
Very high	0	0	0	
High average	0	0	0	
Average	4 (26.7)	2 (100)	2 (15.4)	
Low average	1 (6.7)	0	1 (7.6)	
Very low	0	0	0	
Extremely low	10 (66.7)	0	10 (76.9)	

Data were collected at T-8pre (autistic and gastrointestinal) and T0 timepoint (neuropsychological) and compared between participants according to their sex (females vs. males)

^a^Differences between sex were compared using Mann–Whitney *U*-test (ATEC and GSI score) and Fisher exact test (behavior severity, GI problems and intelligence scale)

^b^Severity of autistic behaviors was classified in 3 categories according to the ATEC scores: mild (score < 49), moderate (score 50–79) and severe (score ≥ 80) according Mahapatra et al*.* [78]

^c^GI problems were determined in participants based on GSI score

^d^WISC-V and IV batteries were used to evaluate intelligence: Extremely high (score ≥ 130); very high (score: 120–129); high average (score: 110–119); average (score: 90–109); low average (score: 80–89); very low (score: 70–79); extremely low (score: 50–69)

*ASD* autism specter disorder, *GI* gastrointestinal, *GSI* Gastrointestinal Severity Index

Yes: total GSI questionnaire score was ≥ 4

### Probiotic product acceptability and safety

All participants (100%) accepted the taste and the consumption of the probiotic beverage (Table 3).

**Table 3:** Description of acceptability, safety, and feasibility data

Parameters	Success	Study results	
	Threshold (%)	Rate (%)	N measured/N expected
Acceptability	> 50	100^a^	22/22
Safety	> 60	100^b^	22/22
Feasibility			
Recruitment rate	> 50	60	23/38
Drop-out rate	< 35	4.35^c^	1/23
Compliance rate (probiotics)	> 50	100^d^	22/22
Completion rate			
*Tests*	> 50		
EEG		86.3	57/66
Actigraphy		94.4	
Blood tests		95.4	42/44
Stool sample		89.2	99/111
Questionnaires^e^	> 80	Mean: 84.6	
ATEC		93.7	104/111
SCQ		93.7	104/111
GSI		93.7	104/111
Questionnaire on usual sleep schedules		93.7	104/111
CSHQ		93.7	104/111
BRIEF		68.5	76/111
24H-R		53.0	59/111
3-day food records		86.5	96/111
Neuropsychological evaluation	> 80	56.8	25/44

^a^Probiotic product was mixed with orange juice for 2 participants and with drinkable yogurt for 1 participant

^b^Probiotic beverage was considered safe when the adverse effects of probiotics were not observed in 60% of participants

^c^One participant dropped out of the study after the T-8pre timepoint and was not taken into consideration in the calculation of success rates of the subsequent visits

^d^To be compliant, participants had to consume the probiotic product at least 6 out of 7 days per week for the 14-week supplementation period

^e^The mean questionnaire completion rate is based on the average completion rate of the 8 questionnaires. The number of questionnaires completed by parents out of the total number of questionnaires is indicated. The pre-established success thresholds are adapted from [87]

*EEG* electroencephalogram, *ATEC* Autism Treatment Evaluation Checklist, *SCQ* Social Communication Questionnaire, *GSI* Gastrointestinal Severity Index, *CSHQ* Children’s Sleep Habit Questionnaire, *BRIEF* Behavior Rating Inventory of Executive Function, *24H-R* 24-h recall

In 3 cases, parents had to mix the product with another food item: orange juice (*N* = 2), or yogurt (*N* = 1). No adverse events requiring discontinuation of probiotic supplementation were reported. Constipation was reported in 3 participants for a duration of 2 days and in one participant for 3 days. All these 4 participants were known to suffer from occasional constipation.

### Feasibility of the study protocol

The feasibility thresholds for the study measures are described in Table 3. The study drop-out rate was 4.25%, as one participant did not complete the study for logistical considerations. This participant was considered for the calculation of data collected at T8-pre (*N* = 23), but not for the other 4 time-points (*N* = 22). Compliance rate for consuming the probiotic during the study was 100% (at least 6 of 7 days a week). In few cases, the probiotic product was consumed only 6 of 7 days a week for a total of 3 (*N* = 3), 2 (*N* = 4) and 1 (*N* = 2) weeks. Blood tests were performed on 95.4% of the scheduled procedures. However, at 4 time-points related to 4 different participants, blood was not successfully collected (totally or partially) for technical reasons (i.e. presence of small or deep veins). Since the procedure was accepted by the children and performed nonetheless, these data were considered positive as to their feasibility. Also, 89.2% of stool samples were collected. Of the non-completed samples, 2 samples were not stored properly and 10 were not provided by parents. The EEG tests were performed in 86.3% of cases. Five participants were unable to complete 9 EEG tests: 2 participants did not perform the test at T0 and T14 (4 tests), one was unable to complete it at T14 and T22 (2 tests), one was unable to do it at T0 (one test) and 2 participants were unable to complete it at T22 (2 tests). The actigraph was worn for 42 periods of 24 h for the entire duration of the test (7 consecutive days). Two participants wore the actigraph for only 5 out of 7 days on one occasion each. In total, the actigraph acceptance rate was 94.4%. Parents were able to complete 84.6% of all questionnaires. The completion rates were inferior to the pre-defined threshold for the BRIEF questionnaire (68.5%) and the 24-HR (53.0%). The completion rate of the neuropsychological evaluation using the WISC-V and WISC-IV batteries was 56.8%. The neuropsychological evaluation could not be performed on 7 non-verbal children, as the test requires children to be verbal, and one verbal child experienced significant stress during testing at both T0 and T14 visits. Three other participants did not complete the neuropsychological evaluation at T0 but successfully completed it at T14.

### Fortuitous discovery

During this study, incidental findings were identified in some children. Cerebral abnormalities were detected by EEG at T0 (confirmed at T14 and T22) in 3 participants (13.6%), including one participant with a known diagnosis of epilepsy. These children were referred to the Neurology Department at CHU Sainte-Justine for follow-up after confirming the abnormality with a neurologist. Also, hs-CRP levels > 1.0 mg/L were detected in 2 children (9.1%) for both at T0 and T14 (5.6 mg/L at T0 and 7.8 mg/L for one child and 28 mg/L at T0 and 30 mg/L at T14 for the other). Parents were informed and follow-up by their pediatrician or family doctor was recommended.

### Preliminary results on the impact of the probiotic beverage on behavioural and GI symptoms

**Fig. 3:**
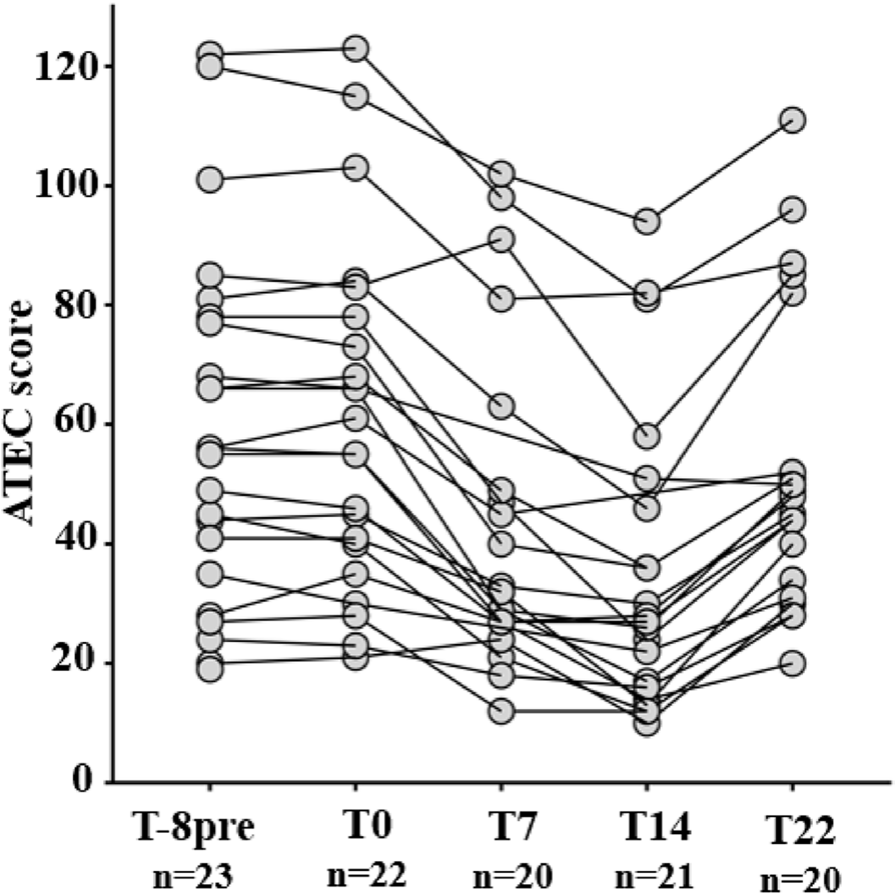
ATEC scores of participants at each study timepoint. The ATEC questionnaire was completed at each visit by parents. Gray circles indicate the ATEC score of 1 participant. ATEC: Autism Treatment Evaluation Checklist

After 14 weeks of intervention, there was a reduction in the ATEC score for all participants, reflecting an improvement in autistic behaviors (Fig. 3).

**Fig. 4:**
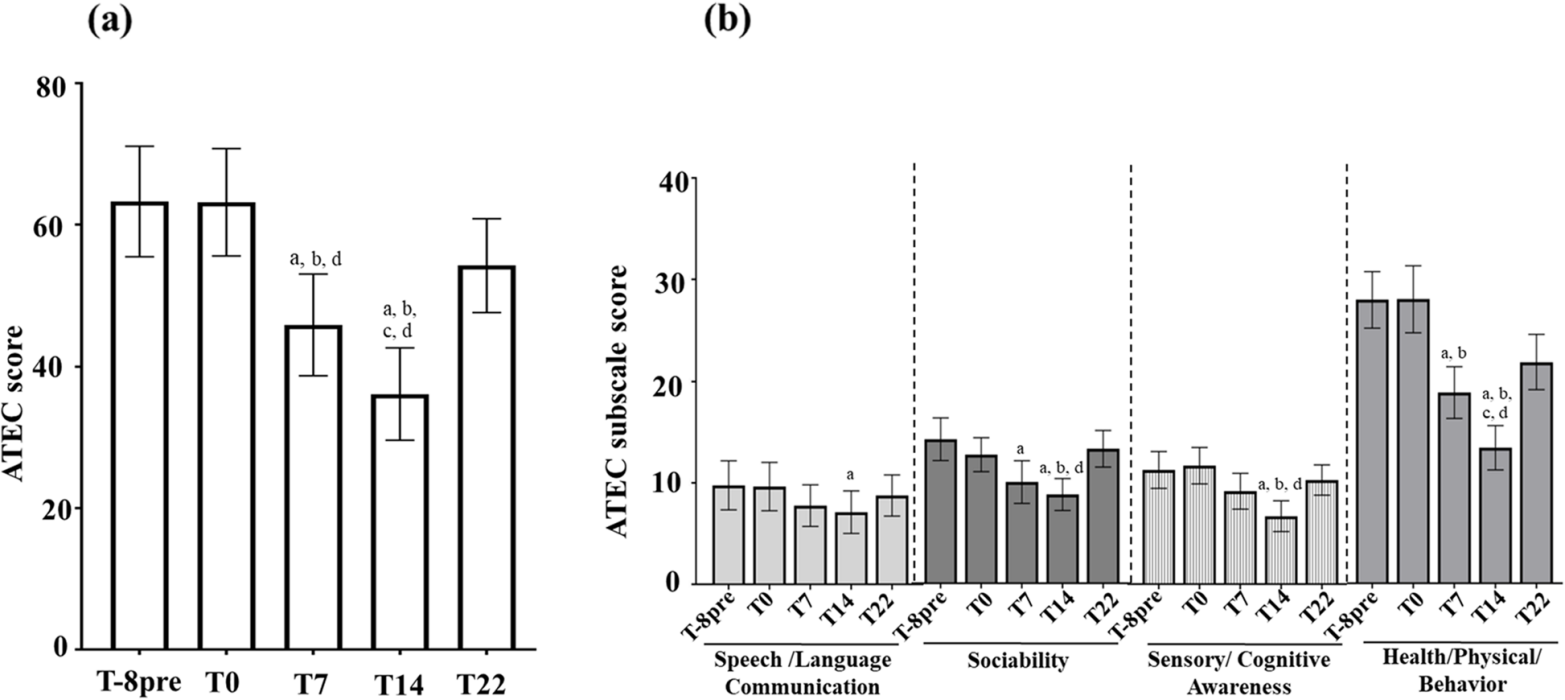
Change of the ATEC and its subscale scores during study. Mean (**a**) ATEC score and (**b**) subscale score was compared across the 5 time-points using repeated measure ANOVA in *N* = 17 participants. *Post-hoc* analysis was conducted for pair-wise comparison between time-points using Bonferroni adjustment for multiple testing. Data represent means with standard error at each time-point. ^a^*P* < 0.05 compared to T8pre; ^b^*P* < 0.05 compared to T0; ^c^*P* < 0.05 compared to T7; ^d^*P* < 0.05 compared to T22. ATEC: Autism Treatment Evaluation Checklist

The mean ATEC total score decreased by 27.1 ± 3.2 points (*P* < 0.001), which represents a 42.8% reduction (Fig. 4a).

Scores for the 4 ATEC domains were also lower after supplementation, with the greatest decline observed in the health/physical/behavior sub-score (−14.6 points ± 2.2) (Fig. 4b).

**Fig. 5:**
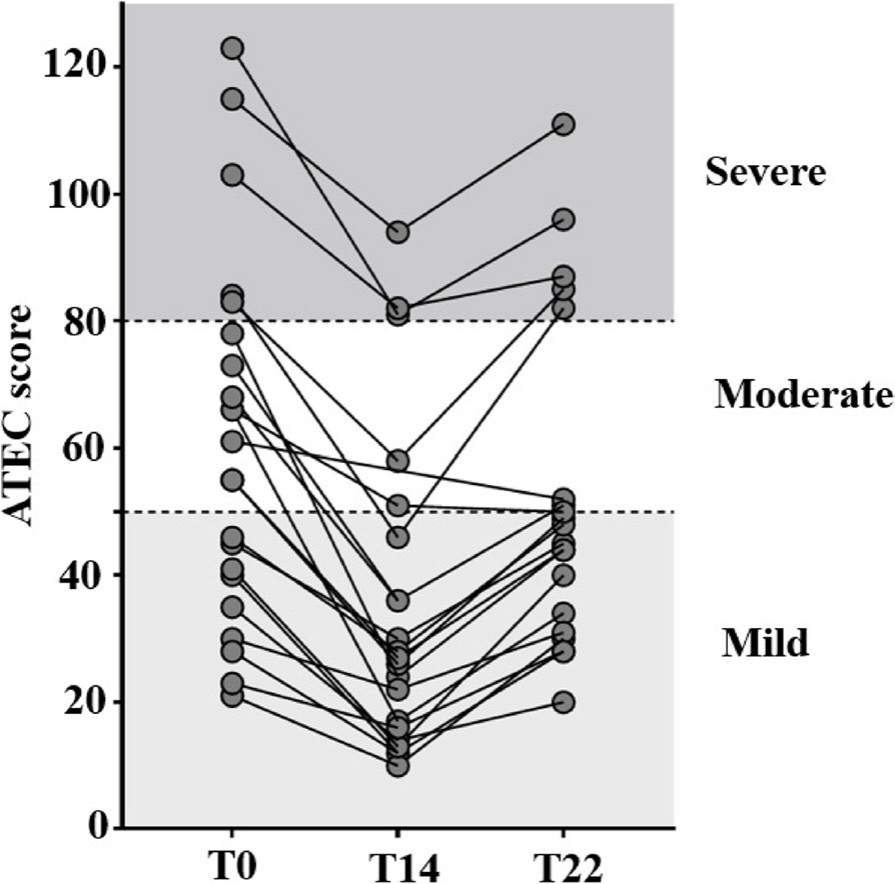
Severity of autistic behaviors of participants during study. Classification of autistic behaviors was assessed for each participant before (T0), at the end (T14) and after (T22) treatment. Severity of autistic behaviors was classified in 3 categories according to the ATEC score: mild (score < 49), moderate (score 50–79) and severe (score ≥ 80) according Mahapatra et al*.* [59]. ATEC: Autism Treatment Evaluation Checklist

According to ATEC score classification, 6 participants initially classified with moderate autism behavior were, after treatment, in the mild category, and 3 participants initially classified with severe autism behavior were classified as moderate thereafter (Fig. 5).

**Fig. 6:**
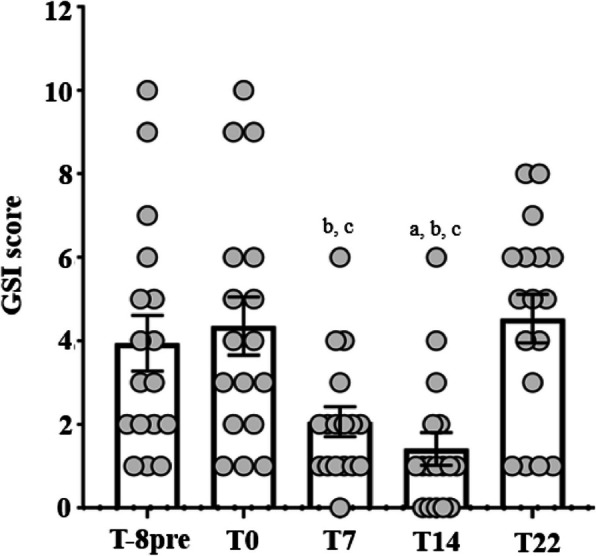
Evolution of the GSI score during study. Mean GSI score was compared across the 5 time-points using Friedman test. *Post-hoc* analysis was conducted using Wilcoxon test for pairwise comparison using Bonferroni adjustment for multiple testing data represents mean with standard error of the 17 participants at each time-point. ^a^*P* < 0.05 compared to T8pre; ^b^*P* < 0.05 compared to T0; ^c^*P* < 0.05 compared to T22. GSI: Gastrointestinal Severity Index

**Fig. 7:**
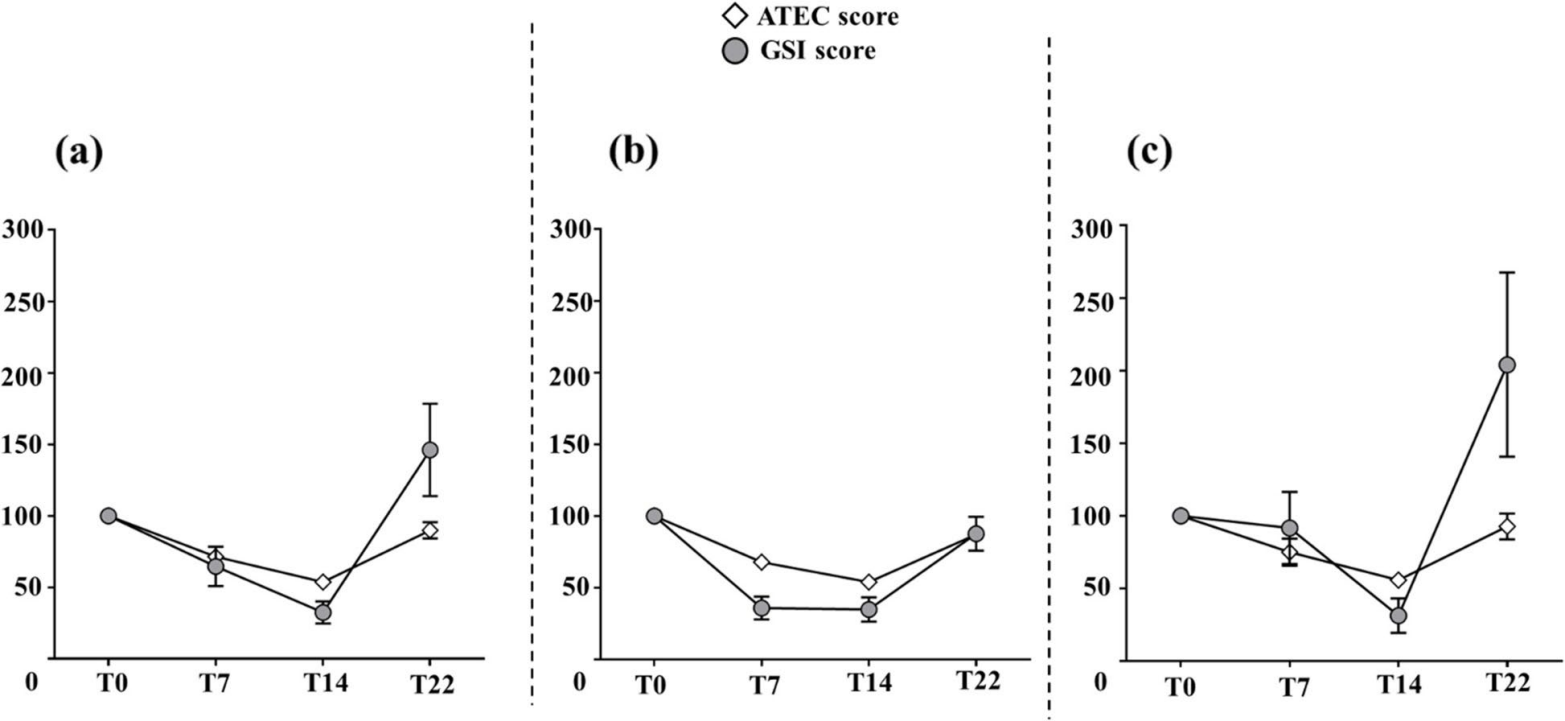
ATEC and GSI scores during study. Mean ± standard error (error bars) of baseline-corrected data of ATEC and GSI scores during (T7 and T14) and after wash-out (T22) are represented as percentage of the T0 score for: **a** all participants (*N* = 17) who completed GSI and ATEC questionnaire at each time-points; **b** participants with severe GI problem (*N* = 13); and (**c**) participants without severe GI problem (*N* = 9). GI problems were classified as severe if GSI score ≥ 4. ATEC: Autism Treatment Evaluation Checklist. GSI: Gastrointestinal Severity Index

Interestingly, 8 weeks after stopping probiotic supplementation (T22), mean ATEC and GSI scores had returned to their initial levels (Figs. 4a, 6 and 7a).

**Fig. 8:**
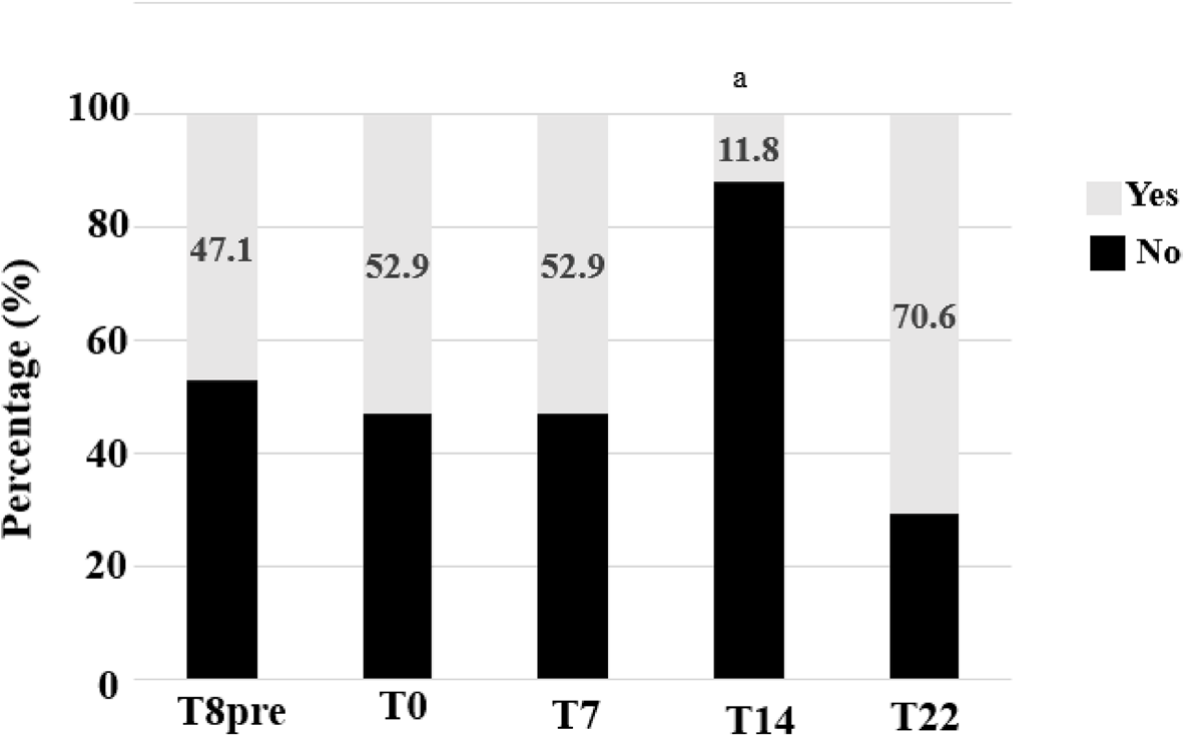
Severity of gastrointestinal symptoms during study. Proportion (%) of participants with severe and non-severe GI problems was assessed at each time-points of the study. ^a^*P* < 0.01 compared to T22 as the difference in proportions across the 5 time-points was compared using Cochran Q test and *post-hoc* analysis was performed using McNemar test for pairwise comparison between time-point with false discovery rate correction for multiple testing. GI problems were classified severe if GSI score ≥ 4. GSI: Gastrointestinal Severity Index

When the cohort was stratified according to the presence of severe GI problems (GSI score ≥ 4), the same tendency in the evolution of ATEC and GSI scores was observed in both groups (Figs. 7b and c). Also, the proportion of participants with severe GI problems was lower at the T14 visit, compared to after the wash-out period (T22) (11.8% vs. 70.6%, *P* < 0.01) (Fig. 8).

**Fig. 9:**
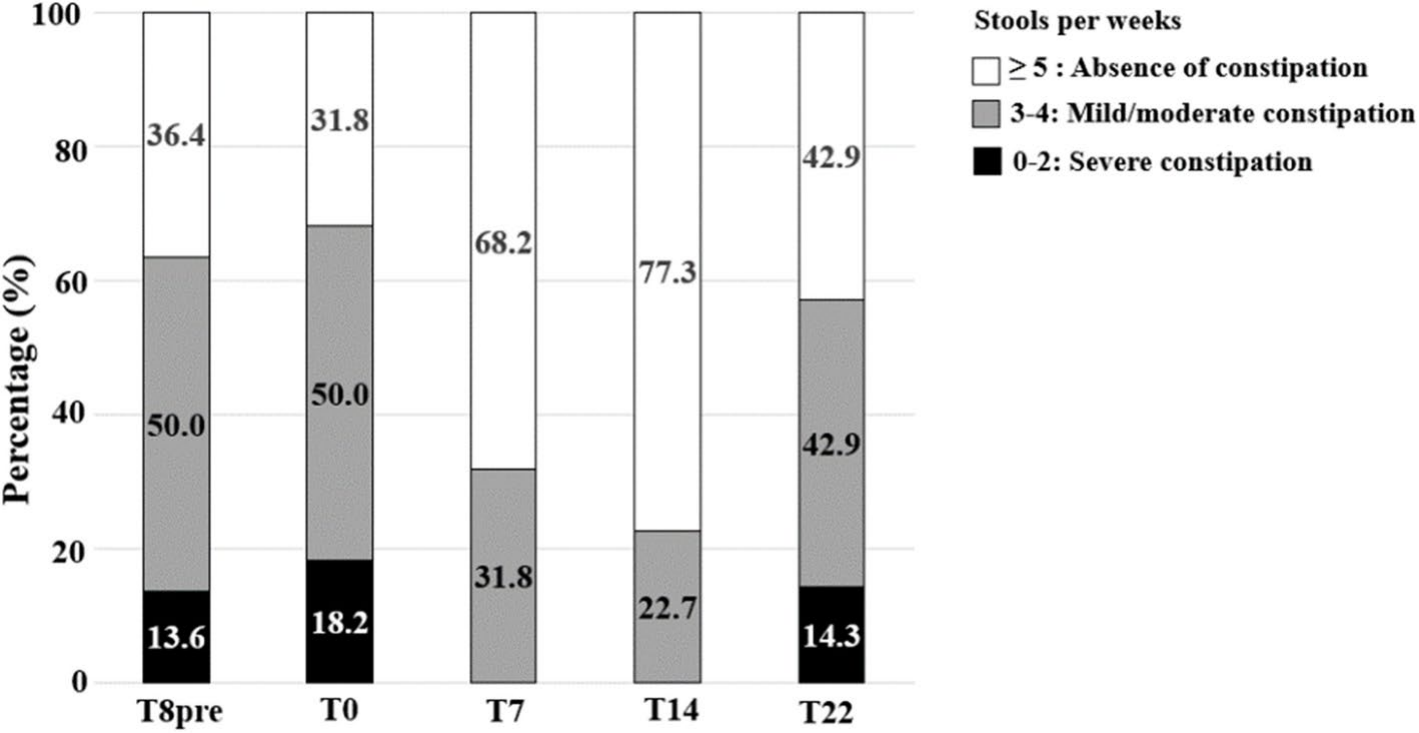
Proportion of participants with and without constipation at study time-points. Proportion (%) of participants with constipation was assessed at each time-points of the study based on the first question of GSI questionnaire. Constipation was identified in participants having 3–4 stools/week (mild/moderate constipation) and 0–2 stools/week (severe constipation) based on the first question in GSI questionnaire. Participants reporting ^3^5 stools per week were considered not constipated

At recruitment (T8-pre), 15 participants (65.2%) had constipation. After probiotic supplementation (T14), constipation was still present in only 5 children (22.7%) but was deemed less severe (Fig. 9).

## Discussion

The findings of this study confirm the acceptability, safety, and feasibility for autistic children to consume a probiotic beverage (Bio-K +) and their ability to undergo the proposed testing and evaluations. They also provide preliminary data supporting a possible positive impact on autistic behaviors and GI symptoms.

Initially, the recruitment of 30 children with a diagnosis of ASD was planned. However, the interim analysis showed that the study objectives were largely achieved earlier and, recruitment was ended after the 23rd participant. In doing so, we avoided unnecessarily exposing children to procedures and related stress, and we optimized the use of resources.

The recruitment process for the study was relatively easy and respected the planned timeline. Retention rate was high, as only one participant dropped out. Many initiatives were deployed to ensure success. At recruitment, parents were thoroughly informed of the protocol, procedures and the estimated time and effort to be invested if accepting to participate in the study. They were allowed sufficient time to think about it and consult their child’s physician if needed. Throughout the study, the research team was available to answer parents'questions by phone or email and measures were adopted to accommodate them when needed. This approach resulted in engaged and collaborative parents, and the visit schedules were usually respected.

Regarding recruitment strategies, recruitment via social media led to a higher recruitment rate than through medical charts. However, the latter allowed a better selection of participants in line with the eligibility criteria (mainly for age and sex). Based on this observation, for future studies, it is proposed to maintain a mixed recruitment method, i.e. via social media, medical charts and by referral from professionals.

Although the sample size was relatively small, the composition of the cohort minimally reflects the socio-demographic portrait of autism reported in the scientific literature. The proportion of females vs. males (30%) in our cohort is representative of the autistic population in Canada. As a matter of fact, the 2019 CHSCY report [3] documented that 29% of children with a diagnosis of ASD aged 1 to 11 years are females [3]. In addition, 30.4% of participants were non-verbal, of whom 57.1% were females. Accordingly, in the literature, 25% to 50% of individuals with a diagnosis of ASD are non-verbal and had not developed functional language [88–92]. In our cohort, 17.4% of participants had a sibling with an ASD diagnosis. In the literature, the sibling recurrence rate, which is defined as the probability for a child with a diagnosis of ASD to have at least one sibling with the same diagnosis, has been estimated to be between 6.1% and 18.7% [93–95].

GI dysfunction has been reported in 9–91% of individuals with ASD [40, 96–101] and reviewed in [102]). In our study, 91% of participants had at least 1 GI problem, 65.2% of which had severe symptoms. Also, 65.2% and 21.7% of participants had constipation and diarrhea, respectively. Constipation is reported to be the primary GI comorbidity in individuals with a diagnosis of ASD [45, 103] and chronic constipation is typically the most common GI disorder, encountered in up to 80% of children with a diagnosis ASD [41, 103–106]. Alternating constipation and diarrhea is also present in this population [107].

In this study, compliance with probiotic supplementation was 100%, with no development of adverse effects requiring discontinuation of supplementation by the participant. Arnold et al*.* had also demonstrated good compliance (97%) with no serious adverse effects in autistic children given the Visbiome™ probiotics for 8 weeks [66]. One aspect that was evaluated was the use of validated questionnaires as tools to capture data with the targeted population. When assessing the impact of an intervention on autistic behaviors and other comorbidities in children with a diagnosis of ASD, different questionnaires can be considered. Not all questionnaires are appropriate for all populations as their characteristics vary. In this study, the threshold for success was reached for all questionnaires and tests, except for those for the neuropsychological evaluation, the BRIEF questionnaire and the 24-h recall. It is concluded that the neuropsychological evaluation using the WISC-V and WISC-IV batteries, and the BRIEF questionnaire were found not adapted for our population as they include questions about language and tasks requiring communication, and that some of our study participants were non-verbal. For future study, we recommend using a non-verbal IQ test, such as the Leither 3 test, which is designed to assess non-verbal cognitive, memory and attention abilities in individuals without language skills [108]. However, we considered that the 24-h recall should remain but would necessitate closer follow-up from the research team.

Several studies demonstrated a significant effect of the intervention with probiotics in individuals with a diagnosis of ASD [109–114] and reviewed at [64]). However, other studies were inconclusive (reviewed at [115]). These studies have led to promising results, but methodological issues (small sample size, non-standard measures, incomplete reporting, lack of placebo) preclude demonstrating an impact of probiotics. In addition, the 2 RCT carried out [63, 66] used the same probiotic product (Visbiome™, containing 8 bacterial strains). One study evaluating the impact of an 8-week supplementation in 13 autistic children suffering from GI symptoms did not achieve significant results on GI symptoms [66]. The other study evaluated the impact of a 6-month supplementation on a severity score for ASD (ADOS calibrated severity score) (*N* = 31 in the probiotics and *N* = 32 in the placebo arm) did not find an effect of treatment on the primary outcome. Nonetheless, secondary analyses revealed a statistically significant decrease in total ADOS score in a sub-group of children with GI symptoms and an improvement in GI symptoms. Of note, the ADOS score is not validated to evaluate response to treatment (reviewed in [116]). At this point, while there is existing weak evidence of the benefit of probiotic preparations in autistic children, this needs to be formally confirmed with a well-conducted RCT with placebo.

While it must be noted that the design of our safety and feasibility study does not allow for a scientifically sound evaluation of an intervention impact (due, for example, to the absence of a placebo group), it led, nonetheless to interesting preliminary data. Notably, a beneficial impact of the probiotic supplementation on the total ATEC score, its 4 sub-categories and on the GSI score was found. For comparison, 5 non-randomized studies evaluating the impact of probiotics on ATEC and/or GSI scores are summarized in Table 4 (Additional File).

In these studies, the reduction of the ATEC scores following probiotics supplementation ranged from 7.5% to 32.4%. The 42.8% reduction observed in our study suggests a potentially greater ability than other probiotic formulations to manage certain behaviors associated with autism. However, while changes in scores were observed after supplementation, for most participants, ATEC scored remained within the same classification range 8 weeks after stopping probiotic supplementation compared to baseline. This reaffirms that the probiotic beverage used in this study is not intended as a treatment, but as a supplement that could help alleviate some of the symptoms associated with ASD. Besides, the reduction in GSI scores reported in the literature is similar to what was found in our study in our study (66% reduction).

The pathogenesis of GI disorders associated with ASD is not yet fully understood. Here, preliminary results suggest a correlation between ASD and GI symptoms. The improvement or disappearance of constipation could be a consequence of the intestinal microbiota normalization following probiotic treatment. GI abnormalities may be a manifestation of an underlying inflammatory process, which is hypothesized to be related to intestinal dysbiosis, or simply reflect interoceptive hyperreactivity [117, 118]. A previous study realised by Preston et al. [72] concluded that the capacity of the probiotic beverage to improve the quality of life of study participants, including dysphoria, health anxiety, food avoidance disorder and social interaction, could be mediated by restoration of gut microbiota.

In our study, sleep disorders affected 91.3% of participants, a number that exceeds what has been reported in the literature reporting between a proportion of children with a diagnosis of ASD with a sleep disorder to be between 44 and 83%. These disturbances can range from bedtime resistance, prolonged sleep delay, long or numerous nighttime awakenings and early morning awakenings [30, 31] and are associated with anxiety, depression, somatic complaints and social problems in children and adolescents with a diagnosis of ASD [119].

Interestingly, the most significant changes were observed in the health/physical/behavior subscale. This suggests that the probiotic beverage may have had the greatest effect on the behaviors and items in this scale, which is not surprising. Several questions in this subscale are related to quality of life, including questions about GI symptoms, sleep, pain sensitivity, anxiety, excitability, attacks and hyperactivity. These features are more likely to be influenced by probiotic supplementation than language, social skills, or sensory seeking behavior. Therefore, by improving GI symptoms and sleep, the probiotic beverage may have a positive impact on behavior, particularly in children who cannot communicate their pain effectively through language. It could also render the child more receptive to other interventions (e.g. ABA, speech) just by being in a better mood.

An important aspect to consider when interpreting this study preliminary data is the potential impact of the placebo effect. As a matter of fact, studies using parent-reported questionnaires may be affected by a significant placebo effect on parent perception [120]. In fact, 50% of the observed effect size of pharmacological and dietary supplement treatment trials could be attributable to the placebo effect [121] hence the value of conducting a randomized controlled trial with placebo to validate the aforementioned preliminary results. Another study limitation is that we did not gather information on participants'involvement in other therapies, such as behavioral therapy, which could have influenced the observed improvements in the ATEC score.

## Conclusion

We conclude that a clinical trial studying the efficacy of the supplementation with a probiotic beverage (Bio-K +) in children with a diagnosis of ASD is acceptable, safe, and feasible. Some minor modifications are proposed to facilitate future clinical studies. Preliminary data support the hypothesis on the efficacy of the probiotic beverage to improve behaviors associated with autism and GI symptoms. Preliminary data seem supporting the hypothesis on the efficacy of the probiotic beverage to improve behaviors and GI symptoms and the relevance to carry out a RCT with placebo. Particular caution is required, given the long history of open-label therapeutic trials showing promising results in autism, which were often followed by disappointing outcomes in more controlled subsequent studies.

## Supplementary Information

Supplementary Material 1.

## Data Availability

No datasets were generated or analysed during the current study.

## Abbreviations

24-HR: 24-Hour Recalls
ABA: Applied Behavior Analysis
ADHD: Attention-Deficit Hyperactivity Disorder (ADHD)
ANOVA: Analysis Of Variance
ASD: Autism Spectrum Disorder
ATEC: Autism Treatment Evaluation Checklist
BRIEF: Behavior Rating Inventory of Executive Function (BRIEF)
CFU: Colony Forming Units
CSHQ: Children’s Sleep Habit Questionnaire (CSHQ)
DSMB: Data and Safety Monitoring Board
EEG: Electroencephalography
ERP: Event-Related Potentials
GI: Gastrointestinal
GSI: Gastrointestinal Severity Index
MTT: Microbial Transfer Therapy
RCT: Randomized Controlled Trials
SCQ: Social Communication Questionnaire
SE: Standard Error
WISC: Weschler Intelligence Scale for Children
