# Supplementary material for: Acceptability and safety of a probiotic beverage supplementation (Bio-K +) and feasibility of the proposed protocol in children with a diagnosis of autism spectrum disorder
Source: J Neurodev Disord. 2025 May 24;17:30. doi: 10.1186/s11689-025-09617-5 (PMC12102953; doi:10.1186/s11689-025-09617-5)
Supplement: Supplementary file 1 — Supplementary Material 1. [file 11689_2025_9617_MOESM1_ESM.docx]

**Additional file:**

**Table 4. Review of the effect of probiotics on ATEC and/or GSI scores**

|  |  |  | **Treatment** |  |  |  | **Effect** | |  |
| --- | --- | --- | --- | --- | --- | --- | --- | --- | --- |
| **Studies** | **N** | **Probiotic strains** | **Concentration** | **Format** | **Duration** | **Score** | **Before** | **After** | **% reduction*** |
|  |  |  |  |  |  |  | Mean (SD) | Mean (SD) |  |
| **This study** | 22 | *L. rhamnosus* CLR2*, L.* | 50 x 10^9^ CFU/day | Probiotic | 14 weeks | ATEC | 63.2 (31.4) | 36.1 (26.8) | 42.8 |
|  |  | *casei* LBC80R and *L. acidophilus* CL1285 |  | drinkable |  | GSI | 4.36 (2.9) | 1.48 (1.6) | 66 |
| *Niu et al* (1) | 37 | N/S | N/S | N/S | 4 weeks | ATEC | 67.1 (N/S) | 59.0 (N/S) | 12.1 |
|  |  |  |  |  |  | GSI | 2.26 (N/S) | 0.84 (N/S) | 62.8 |
| Tharawadeep-himuk *et al*. (2) | 10 | *L. rhamnosus,* *L. paracasei* and *B. Longum* | 20x10^9^ CFU/day |  | 6 weeks | ATEC | 65.2 (30.66) | 50.6 (19.44) | 22.4 |
| Wang *et al*. (3) | 36 | *L. rhamnosus* HN001, *B.* | 10^10^ CFU/day | Powder | 14 weeks | ATEC | 85 (5.72) | 59.3 (5.52) | 30.3 |
|  |  | *lactis* BL-04, and *L. paracasei* LPC-37+ **FOS** |  |  |  | GSI | 4.88 (0.43) | 1.44 (0.38) | 70.5 |
| Shaaban *et al*. | 30 | *L. acidophilus, L.* | 5 × 10^8^ CFU/day | Powder | 3 months | ATEC | 93.4 (12.8) | 80.8 (10.54) | 13.5 |
| (4) |  | *rhamnosus* and *B. longum* |  |  |  | GSI | 7.23 (1.61) | 3.57 (0.96) | 50.6 |
| Li *et al*. (5) | 21 | *B. longum, L. acidophilus* and *Enterococcus faecalis*  *+* ABA | 2X10^7^ CFU/twice a day; | Powder | 3 months | ATEC | 84 (27) | 57 (23) | 32.4 |
| West *et al*.(6) | 33 | *L. acidophilus, L. casei, L. delbruecki, B.* *longum, B. Bifidum*  + Del-Immune V | 1 X 10^8^ CFU | Capsules | 6 months | ATEC | - 1. (N/S) | 58.3 (N/S) | 20 |

*Note*. *% reduction in ATEC score before/after treatment was calculated on the basis on the results provided in the respective articles.

N/S: not specified. N/A: not measured. CFU: colony forming unit. FOS: fructo-oligosaccharide. ABA: applied behavior analysis.

1. Niu M, Li Q, Zhang J, Wen F, Dang W, Duan G, et al. Characterization of intestinal microbiota and probiotics treatment in children with autism spectrum disorders in China. Frontiers in neurology. 2019;10:1084.

2. Tharawadeephimuk W, Chaiyasut C, Sirilun S, Sittiprapaporn P, editors. Preliminary study of probiotics and kynurenine pathway in autism spectrum disorder. 2019 16th International Conference on Electrical Engineering/Electronics, Computer, Telecommunications and Information Technology (ECTI-CON); 2019: IEEE.

3. Wang Y, Li N, Yang J-J, Zhao D-M, Chen B, Zhang G-Q, et al. Probiotics and fructo-oligosaccharide intervention modulate the microbiota-gut brain axis to improve autism spectrum reducing also the hyper-serotonergic state and the dopamine metabolism disorder. Pharmacological research. 2020;157:104784.

4. Shaaban SY, El Gendy YG, Mehanna NS, El-Senousy WM, El-Feki HS, Saad K, et al. The role of probiotics in children with autism spectrum disorder: A prospective, open-label study. Nutritional neuroscience. 2018;21(9):676-81.

5. Li Y-Q, Sun Y-H, Liang Y-P, Zhou F, Yang J, Jin S-L. Effect of probiotics combined with applied behavior analysis in the treatment of children with autism spectrum disorder: a prospective randomized controlled trial. 2021.

6. West R, Roberts E, Sichel L, Sichel J. Improvements in gastrointestinal symptoms among children with autism spectrum disorder receiving the Delpro® probiotic and immunomodulator formulation. J Prob Health. 2013;1(1):1-6.
